# Association between Non-Verbal Intelligence and Academic Performance of Schoolchildren from Taza, Eastern Morocco

**DOI:** 10.3390/jintelligence10030060

**Published:** 2022-08-19

**Authors:** Said Bouchefra, Amal Azeroual, Hassan Boudassamout, Khalid Ahaji, Abdelhakim Ech-chaouy, Abdellatif Bour

**Affiliations:** 1 Laboratory of Biology and Health, Team of Nutritional Sciences, Food and Health, Faculty of Sciences, University Ibn Tofail, 14000 Kenitra, Morocco; 2 M2CS, Research Center STIS, National Graduate School of Arts and Crafts Rabat (ENSAM), Mohammed V University, B.P:8007.N.U Rabat, Morocco; 3 Center of Guidance and Planning, Hay Ryad (COPE), B.P:8007.N.U Rabat, Morocco

**Keywords:** academic achievement, cognitive ability, non-verbal intelligence, schoolchildren, Raven’s Standard Progressive Matrices

## Abstract

Interest in identifying factors influencing educational success is growing. It is often observed that a group of students share the same external variables (school environment) yet have different results, which states that individual variables have more impact on the determination of academic performance. Therefore, the present study aimed to substantiate this fact by investigating the association between non-verbal fluid intelligence and academic performance in a population of schoolchildren in Eastern Morocco. The investigation was a cross-sectional study based on a self-administered questionnaire. Items included the standard Raven’s progressive matrices. Students’ grades were collected from the administrative offices of the visited schools. Significant and positive correlations between the non-verbal intelligence scores and the school results were found: for the general average, the correlation was 0.574; for the school subject French, the correlation coefficient was 0.475; and for mathematics, we found a relatively low coefficient of 0.381. Non-verbal fluid intelligence significantly and positively predicted academic performance (β = .574, *p* = .000). These results call for policymakers to implement the use of intelligence tests with school directors and teachers as a diagnostic tool to guide support efforts for low-achieving children and even to create pilot classes for the best-performing students.

## 1. Introduction

Poor academic performance is a common issue in all countries in the world, especially developing ones (OECD 2021). Standardized assessments of cognitive performance are used in different fields, especially in education. One of the reasons for the widespread use of these assessments is their supposed ability to predict success (Koch et al. 2021). Several studies have confirmed the association between intelligence, on the one hand, and educational success (Deary et al. 2007; Downey et al. 2014; Mayes et al. 2009), nutritional status (Akubuilo et al. 2020), professional success (Damian et al. 2015), success in relationships (Aspara et al. 2018), and health (Wrulich et al. 2014), on the other hand, hence the growing interest in evaluating neurocognitive performance, in particular intelligence, using tests dedicated to this purpose. There are currently many intelligence tests available, and the most widely used are the Wechsler scales, the British Ability Scales, Raven’s Progressive Matrices, and the Mill Hill Vocabulary Scale (van de Vijver and Leung 1997). Many psychologists consider Raven’s Progressive Matrices to be the purest measure of general intelligence, uninfluenced by cultural and educational factors (Martínez-Rubio 2013; Rushton et al. 2004). Raven’s Progressive Matrices is a non-verbal reasoning test intended to assess abstract and cognitive functioning, spatial reasoning, analogy ability, and problem-solving ability. The test is also a good measure of fluid intelligence, which is the ability to reason and solve problems based on new information only and without relying on previously acquired knowledge or skills. The test is available in three different forms: standard, colored, and advanced. The standard version is the original form of the test published in 1938. Usually, the advanced form is the one given to adults, although the colored version can be used for people with mental or physical disabilities that would make the black and white versions too difficult to pass. The questions are all non-verbal.

Numerous studies have reported high correlations between intelligence level and academic performance, varying from 0.50 to 0.70 (Lynn and Vanhanen 2012; Sternberg et al. 2001; Weber et al. 2013). In a recent meta-analysis summarizing 74 studies that explored the predictive power of intelligence on academic achievement, Katharina et al. reported mean correlations between intelligence (r = .44) and academic achievement (Kriegbaum et al. 2018). Other research using modeling as a statistical tool to predict academic performance also identifies intelligence as a relevant predictor of academic success (Colom and Flores-Mendoza 2007; Karbach et al. 2013; Laidra et al. 2007). In contrast to correlational studies, intervention studies with rigorous exploratory methods (e.g., randomized controlled trials) can provide convincing evidence of relationships between cognitive skills and academic achievement. Most studies have focused on the training of memory and executive function and found that these skills can be used to improve academic performance (Jacob and Parkinson 2015).

Researchers have also examined sex differences in intelligence (Dunkel and Madison 2022; van der Linden et al. 2017) and found a significant difference between girls and boys (Dutton et al. 2018; Flores-Mendoza et al. 2013); other authors found no significant difference in average intelligence scores between the two sexes (Deary et al. 2003).

The performance of an education system is measured by the results obtained by learners on various international tests. Among these tests are the surveys administered by the International Association for the Evaluation of Educational Achievement (IEA). Since 1958, the IEA has measured student achievement in subjects such as math and science TIMSS (Trends in Mathematics and Science Study) and PIRLS (Progress in International Reading Literacy). However, Morales-Vives et al. (2020), reporting on the study conducted by Hoffman and Lowitzki (2005), revealed that the use of student grades to assess academic achievement shows that high school grades are a useful measure and stronger predictors of achievement than standardized tests (Morales-Vives et al. 2020).

Morocco is one of the developing countries with relatively low levels of student achievement. TIMSS 2019 was attended by 64 countries. Morocco participated for the third consecutive time (2011, 2015, 2019) in this survey. For Moroccan 4th grade students who participated in the TIMSS assessments, the score is 383 in mathematics, far from the midpoint of the TIMSS scale (500). They are among the five lowest countries with Kuwait (same score), South Africa (374), Pakistan (328), and the Philippines (297) (Mullis et al. 2019). The same results were found for the PIRLS 2016 study which stated that Morocco, which is in the 48th position, occupies, alongside Egypt (49th) and South Africa (50th), one of the last three places in the ranking (Mullis et al. 2017). Therefore, the objective of this manuscript was to review the factors that influence students’ academic performance in mathematics and their first foreign language. From this perspective, the research attempted to describe and compare the performance gaps observed among learners in different schools, in an attempt to understand the role of cognitive performance in determining academic achievement.

These controversial results underline the questions that our study attempted to answer, namely: What is the level of non-verbal intelligence of schoolchildren in Eastern Morocco? Is the intelligence score correlated with academic achievement? Is there a sex effect in the assessment of academic achievement and intelligence? Few research studies have been conducted based on Raven’s Progressive Matrices test on the Moroccan population. Thus, this research addressed these questions through this test.

## 2. Materials and Methods

### 2.1. The Study Design

The present research is a cross-sectional study based on a self-administered questionnaire containing, in addition to the sixty standard progressive matrices of Raven, sociodemographic information concerning the student, namely their sex, age, and school level. Eight public and three private schools were randomly selected, and then classes were randomly drawn from each school. The survey was conducted from February to April 2022. The sample comprised a total of 528 schoolchildren. The questionnaire was distributed by a previously trained team and completed individually in the classrooms within each school visited by the students. The duration of response to the test was limited to 45 min as indicated in the manual of the standard progressive matrices of Raven.

#### 2.1.1. Variables Measured during the Interview

- Intelligence Assessment

**Figure 1:**
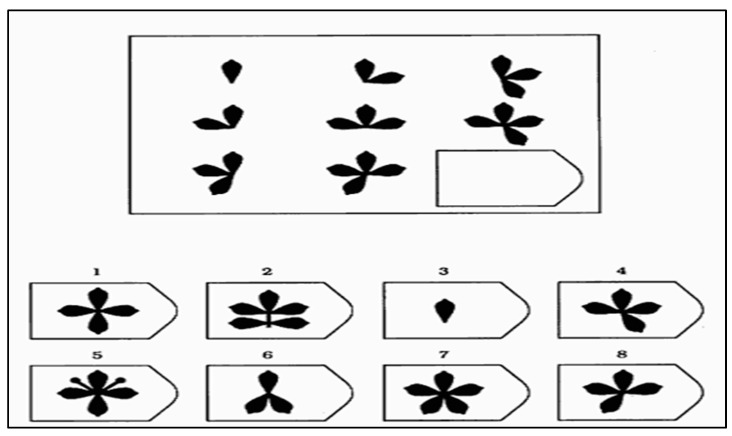
An example of Raven Progressive Matrices Task (Series C). Note: The forms from 1 to 8 are the possible answers, only one of them is correct.

The original form of the Raven Standard Progressive Matrices (RSPM), first published in 1938, was the tool to measure non-verbal intelligence. The test consists of five sets (ranging from A to E) of 12 items each, with the items in each set becoming increasingly complex and requiring greater and greater cognitive ability to analyze and solve the problems. All items are presented in black ink on a white background. Figure 1 shows an example of a spot in the C series, which is based on the principle of rearranging the elements in the matrix. It is necessary to find the grouping which operates in horizontal as well as vertical positions. The enrichment of the figures with additional elements follows a precise principle; having discovered which one, it will be possible to recover the missing figure which is 7.

- Academic Performance Assessment:

For the purpose of this study, grade point average (GPA) was used as an indicator of academic achievement. GPA is a number representing the average value of final grades accumulated in the various school subject tests over time. A student’s academic performance assessment was based on his or her results on the mid-year exams. Two academic subjects were considered, French as a first foreign language and mathematics, and each academic subject was scored on a scale of zero to twenty points, the same as for mid-year GPA. As a result, three variables were collected for each student: the GPAf, which is the grade point average in the first foreign language, the GPAm, which is the grade point average in mathematics, and the GPA, which is the grade point average in all academic subjects combined.

#### 2.1.2. Procedure

Regarding academic performance, students’ grades were collected from the administrative offices of the schools visited. The sample of participants was asked to respond during regular class time to the Raven intelligence test. Participants completed the tests using their names. They received no reward for their participation. However, each student received his or her raw score after taking Raven’s progressive matrices test.

### 2.2. Ethical Considerations

All precautions according to the Declaration of Helsinki (Finland, June 1964) were taken to protect the privacy and confidentiality of the personal information of those involved in the research. Informed consent was obtained from the guardians, who were properly informed of the objectives, methods, and institutional affiliations of the researchers and assured that their responses would be kept anonymous and confidential. Authorization to conduct the survey in public schools in the province of Taza (Morocco) was obtained from the provincial delegation of the Ministry of National Education. The directors of the institutions were informed one week before the visit.

### 2.3. Statistical Analysis

Data analysis was performed using SPSS version 26 software. Quantitative variables were expressed as means and standard deviations, and qualitative variables were expressed as frequencies (percentages). The study used Pearson’s correlation to test the underlying hypotheses.

## 3. Results

### 3.1. Descriptive Statistics

A total of 528 students completed the questionnaire, 43.7% of whom were females. The average age was 14.76 ± 1.92 years. RSPM score appears to vary considerably between students (37.92 ± 11.15). The mid-year average varies moderately (10.89 ± 3.32), even for math (9.38 ± 3.91) and French (7.69 ± 4.12). The results of the descriptive statistics are summarized in Table 1.

**Figure 2:**
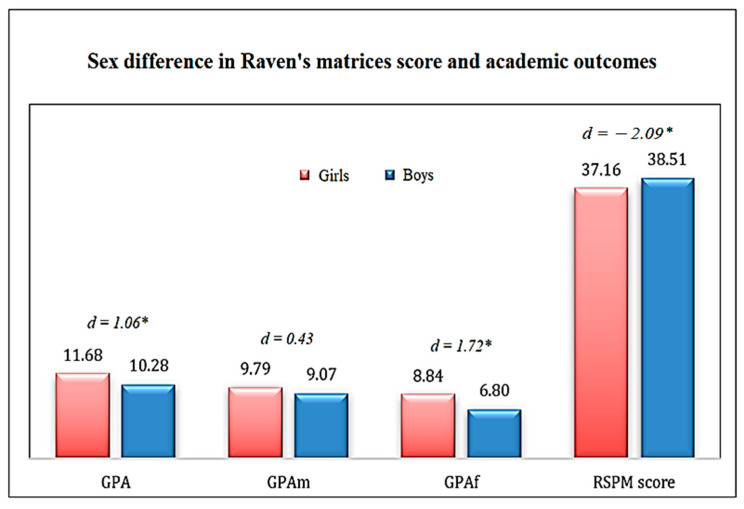
Distribution of participants’ performances by sex. Note: d: differences in mean scores between boys and girls. Negative d value is an advantage score in boys compared to girls, and positive d value is an advantage score in girls compared to boys. The asterisk (*) means a statistically significant *p*-value (*p* < 0.05).

We wanted to compare the difference between the sexes for each variable, namely the Raven score results and the academic performance. A significant *p*-value was observed for the French GPA and the first semester GPA as well as for the Raven matrices score; however, there were no significant results between the two sexes for the mathematics GPA. Indeed, the results show that the boys have an improvement over the girls in terms of intelligence scores and girls excel in French and mid-year GPA. Despite the significant difference between the two sexes (*p*-value < 0.05), the effect size was small for the four variables between the two sexes (r < 0.2). The results of the comparison are presented in Figure 2.

Table 2 shows the corresponding 5th, 10th, 25th, 50th, 75th, 90th, and 95th percentile raw RSPM scores by age groups. We note that the mean RSPM scores generally increase with age.

To situate the results of our study sample in an international context, we used the standardizations from studies conducted in Chile (Queiroz-Garcia et al. 2021), Kuwait (Abdel-Khalek and Raven 2006), the United Kingdom (Raven 1981), Slovenia (Boben et al. 1998), and India (Raven 2000). We compared the median 50th percentile to the previously mentioned studies, and we note that the scores obtained by our students in all age groups are lower than those obtained in the United Kingdom, Slovenia, and Chile and marginally better than those obtained in India. Results are reported in Table 3.

### 3.2. Exploratory Correlations

To answer our main research question which suggests the presence of a positive association between cognitive performance and academic achievement, the correlations between the raw scores of the Raven’s Standard Progressive Matrices (RSPM) test and the grade point averages (GPAs) obtained at the end of the first semester of the academic year were tested. Overall, we found significant and positive correlations between the non-verbal intelligence scores and the school results. For the general average, the correlation was 0.574; for the school subject French, the correlation coefficient was 0.475; and for mathematics, we found a relatively low coefficient of 0.381. Results are summarized in Table 4.

To examine the correlations of the scores by sex, the analysis of the outputs cross-sex was conducted. Girls’ Raven test scores show strong correlations with academic performance compared to boys who have weak correlations with academic performance. Table 5 summarizes the obtained results.

### 3.3. Linear Regression

Given that the exploratory correlation revealed a significant and positive association between non-verbal fluid intelligence and academic performance in our sample, we proceeded with linear regression analysis to verify the explanatory power of this predictor (intelligence score) toward academic performance. GPA was used as the dependent variable. The regression model was significant and explained 33% of the variance in academic performance means (R^2^ = .33 and *p* =.000). Interestingly, non-verbal fluid intelligence significantly and positively predicted academic performance (β = .574, *p* = .000). In other words, when a student’s intelligence score increases by one point, their GPA increases by 17% (B = 0.170). The model obtained shows a large effect size (f^2^ = 0.49) which supports the strong association between the independent variables and the dependent variable (Table 6).

## 4. Discussion

This study aimed to test the associations between non-verbal fluid intelligence and academic performance in a sample of adolescents from Eastern Morocco. Comparing the intelligence scores of the students in our sample obtained in 2022 with the standards of other developed countries dating from the 1990s, which were relatively low, and if we take into account the Flynn effect (Antonio 1988), which dictates an improvement in intelligence scores over the years, the scores obtained by our study population are declared to be more mediocre.

With the objective of investigating the supposed association between non-verbal intelligence and academic performance, correlation tests were conducted. Our results show positive correlations between the intelligence and academic performance of these children. Participants with higher scores on Raven’s Standard Progressive Matrices test demonstrated better academic performance. These correlations are consistent with previous findings showing similar associations between non-verbal fluid intelligence and academic achievement. Indeed, in a meta-analysis including 240 studies, Roth et al. revealed correlation coefficients between intelligence and academic performance ranging from 0.30 to 0.70; we retain those using Raven’s Standard Progressive Matrices (Roth et al. 2015). Agnoli et al. who worked on a sample of 352 Italian schoolchildren reported a correlation coefficient of 0.38, with a sample of 780 participants from Hong Kong (Agnoli et al. 2012), Phillipson reported 0.28 (Phillipson and Phillipson 2012), Axelsson reported 0.5 (Axelsson 2009), and Duckworth et al. found 0.41 (Duckworth et al. 2012). To further examine the underlying hypothesis, we conducted a linear regression analysis in which intelligence was revealed as a predictor of academic achievement by explaining 33% of the variance in academic outcomes. These results are in line with previous studies (Demetriou et al. 2019; Greiff and Neubert 2014; Lechner et al. 2017). Our results demonstrate a stronger association between cognitive performance and the French school subject compared to the association with math. This difference contradicts previous research that stipulates a high correlation between intelligence and science subjects that solicit abstract reasoning (Mathematics and Natural Sciences, as an example) (Ackerman and Heggestad 1997; Lubinski 2020; Spinath et al. 2014) and could be mediated by other factors drawn from the school environment (i.e., the teacher effect, which implies that the teacher can arouse students’ interest and pleasure in learning (Stankov 2013)) or by determinants related to the student themselves since, according to the literature review conducted by R. Su, students excel in the tasks they prefer (Su 2020). Another explanation could be the methodology of academic assessment in language subjects that includes criteria of oral participation and creativity much more than mathematics, which allows for a better assessment of understanding and assimilation.

In agreement with previous studies, our results show sex differences on the standard Raven’s progressive matrices. In our study, the slightly better performance of boys on the RSPM is in agreement with (Lynn and Irwing 2004). These results contradict the results found by Flores-Mendoza et al. (2013), which show better results in cognitive performance tests for girls compared to boys (Flores-Mendoza et al. 2013). The present study indicates that the results obtained by schoolchildren in Eastern Morocco are higher for girls than for boys in the first foreign language and mid-year GPA. There is no significant sex difference in mathematics scores. These results are in line with some studies in the literature that have found higher scores among girls in languages but no significant differences in mathematics (Spinath et al. 2010). Sex differences in academic achievement could be explained, according to (Blair 2002), by the fact that the transition from elementary to secondary school is accompanied by strong demand for behavioral self-regulation, which allows the student to remember and follow instructions and focus on tasks without losing concentration. Therefore, behavior regulation is positively related to the development of positive classroom behavior and academic success (McClelland et al. 2007), and since the girls demonstrated greater motivation and behavioral regulation than the boys, their academic performance was better (Cross et al. 2011).

## 5. Conclusions

The present study was carried out among adolescents aged between 11 and 19 years old and attending school in Eastern Morocco. Firstly, our study was able to highlight the low non-verbal intelligence of children from Eastern Morocco compared to children from socio-economically developed countries. This weakness could influence the children’s school career; thus, it could contribute to the increase in the percentage of school failure and even to an increased rate of school dropout.

Secondly, we were able to demonstrate a moderate correlation between non-verbal intelligence and academic performance. These results call for policymakers to implement the use of intelligence tests by school principals and teachers as a diagnostic tool to guide academic support efforts for low-achieving students and create special classes for high-achieving students.

Finally, the study revealed a significant difference between girls and boys: boys have an advantage in the Raven test, and girls perform well on school exams. The mechanisms behind this difference need to be explored in future research.

Our study provides more evidence regarding the responsibility of cognitive ability in predicting academic performance. However, the differences that exist in terms of correlations across academic subjects (first foreign language and mathematics) are a limitation of our study which highlights the need to introduce all common academic subjects among students for a better understanding of the relationship. Another limitation of our study is the use of a cross-sectional exploration, which does not take into consideration possible fluctuations in academic performance due to many uncontrollable factors (e.g., the health status of subjects that could influence their academic performance during a given semester). Future replication of this work should use a longitudinal investigation spread over long periods. Another issue is that most of the studies reviewed in this manuscript focused on the assessment of academic performance through the grades obtained by students. However, not all teachers use the same criteria to grade their students. For this reason, further studies should be conducted using standardized tests.

## Figures and Tables

**Table 1:** Descriptive statistics.

Variables	Percentage (%)		Mean	SD
Sex	Boys	56.3	-	-
	Girls	43.7	-	-
Age		-	14.76	1.39
RSPM score		-	37.92	11.15
GPAf		-	7.69	4.12
GPAm		-	9.38	3.91
GPA		-	10.89	3.32

Note: RSPM is Raven Standard Progressive Matrices; GPA, GPAf, and GPAm are mid-year grade point average, grade point average in French, and grade point average in math subject, respectively; SD: standard deviation.

**Table 2:** Distribution of SPM scores by age groups in percentiles.

Age Groups							
**Percentiles**	12	13	14	15	16	17	18
95	-	47	47	53	55	50	-
90	43	43	46	52	53	49	-
75	42	41	40	49	48	47	49
**50**	**36**	**34**	**34**	**45**	**44**	**45**	**48**
25	29	27	26	37	31	36	47
10	4	16	14	24	21	28	47
5	4	14	8	19	14	23	47
n	92	67	86	94	40	90	59

**Table 3:** Medians of age-specific SPM scores for Eastern Moroccan students (2022) compared to other populations.

	Age Groups						
**Percentiles**	12	13	14	15	16	17	18
**Our study, Eastern Morocco, 2022**	**36**	**34**	**34**	**45**	**44**	**45**	**48**
Metropolitan Area Chile, 1986, 1987	36	40	45	45	40	52	51
Kuwait, 2006	37	44	43	46	-	-	-
Indian Tribal Areas, 2006	-	23	-	29	-	45	-
United Kingdom, 1979	39	41	43	45	47	48	-
Slovenia, 1998	42	43	44	45	46	48	49

**Table 4:** Correlations between non-verbal intelligence and academic performance outcomes.

RSPM Raw Score	GPAm	GPAf	GPA
**Pearson correlation (r)**	.381 **	.475 **	.574 **
**Significance (Bilateral) (p)**	.000	.000	.000

Note: **: Significance at 0.01.

**Table 5:** Correlations between non-verbal intelligence and academic performance outcomes.

		GPAm	GPAf	GPA
**Boys**	Pearson correlation RSPM Score (r)	.291 **	.475 **	.582 **
	Significance (p)	.000	.000	.000
**Girls**	Pearson correlation RSPM Score (r)	.497 **	.540 **	.612 **
	Significance (p)	.000	.000	.000

Note: **: Significance at 0.01.

**Table 6:** Linear regression analysis with GPA as a dependent variable and RSPM score as a predictor.

	R, R^2^	SE	t	p	B	Beta	f^2^
**Model summary**	.572, .33	-	-	-	-	-	-
**Predictor: RSPM Score**	-	.011	15.984	.000	.170	.572	0.49

Note: SE: standard error. f2: Cohen’s f (effect size).

## Data Availability

Data supporting reported results available upon request.
